# A Two-Phase Stochastic Dynamic Model for COVID-19 Mid-Term Policy Recommendations in Greece: A Pathway towards Mass Vaccination

**DOI:** 10.3390/ijerph18052497

**Published:** 2021-03-03

**Authors:** Nikolaos P. Rachaniotis, Thomas K. Dasaklis, Filippos Fotopoulos, Platon Tinios

**Affiliations:** 1 Department of Industrial Management and Technology, University of Piraeus, 18534 Piraeus, Greece; dasaklis@unipi.gr; 2 Department of Informatics, University of Piraeus, 18534 Piraeus, Greece; filfwt@hotmail.com; 3 Department of Statistics and Insurance Science, University of Piraeus, 18534 Piraeus, Greece; ptinios@unipi.gr

**Keywords:** COVID-19, compartmental models, stochastic dynamic network

## Abstract

From 7 November 2020, Greece adopted a second nationwide lockdown policy to mitigate the transmission of SARS-CoV-2 (the first took place from 23 March to 4 May 2020), just as the second wave of COVID-19 was advancing, as did other European countries. To secure the full benefits of mass vaccination, which started in early January 2021, it is of utmost importance to complement it with mid-term non-pharmaceutical interventions (NPIs). The objective was to minimize human losses and to limit social and economic costs. In this paper a two-phase stochastic dynamic network compartmental model (a pre-vaccination SEIR until 15 February 2021 and a post-vaccination SVEIR from 15 February 2021 to 30 June 2021) is developed. Three scenarios are assessed for the first phase: (a) A baseline scenario, which lifts the national lockdown and all NPIs in January 2021; (b) a “semi-lockdown” scenario with school opening, partial retail sector operation, universal mask wearing, and social distancing/teleworking in January 2021; and (c) a “rolling lockdown” scenario combining a partial lifting of measures in January 2021 followed by a third nationwide lockdown in February 2021. In the second phase three scenarios with different vaccination rates are assessed. Publicly available data along with some first results of the SHARE COVID-19 survey conducted in Greece are used as input. The results regarding the first phase indicate that the “semi-lockdown” scenario clearly outperforms the third lockdown scenario (5.7% less expected fatalities); the second phase is extremely sensitive on the availability of sufficient vaccine supplies and high vaccination rates.

## 1. Introduction

The global spread of SARS-CoV-2, the pathogen that causes the disease COVID-19, has created an infectious disease crisis worldwide and is straining healthcare systems to their limits. The COVID-19 outbreak presents the greatest challenge after World War II. As its repercussions quickly spilled over from healthcare into sectors such as global logistics and trade, travel and tourism, retail, energy, and finance, it has wreaked generalized havoc. By 31 December 2020, 81,947,503 confirmed cases of COVID-19, including 1,808,041 deaths (https://covid19.who.int/?gclid=CjwKCAiArbv_BRA8EiwAYGs23Dd0_laqiTJ8UIMXAnWnWKCI9D_6gQlOsILFa_XMFoVPUPfnRkfxMhoCdi4QAvD_BwE; accessed on 31 December 2020) were reported globally. Greece over the same period recorded 138,850 confirmed cases of COVID-19 and 4838 deaths (https://eody.gov.gr/epidimiologika-statistika-dedomena/ektheseis-covid-19/; accessed on 31 December 2020) for a population of almost 11 million; this performance ranks favorably among advanced countries, but still represents a major national social and economic shock (https://coronavirus.jhu.edu/data/mortality; accessed on 31 December 2020).

In the absence of effective therapies and vaccines until December 2020, countries around the globe adopted Non-Pharmaceutical Interventions (NPIs) to control the spread of the disease and to minimize death rates. NPIs adopted in Greece have included: social distancing and lockdowns, travel restrictions, closure of schools and universities, avoidance of mass gatherings, point of entry screening, teleworking, community-wide containment, iterative contact tracing, quarantining and self-isolation strategies, etc. The development and availability from the start of 2021 of vaccines is a game changer: Several promising vaccine candidates are under development (many in phase 3 of clinical trials) and some have already been licensed [1]. Already by 1 January 2021 the World Health Organization (WHO) listed the Comirnaty COVID-19 mRNA vaccine for emergency use, making the Pfizer/BioNTech vaccine the first to receive emergency validation from WHO since the beginning of the COVID-19 outbreak; approval and roll out of vaccines is proceeding apace.

As vaccines are becoming available, countries are trying to develop exit strategies from the COVID-19 outbreak. Such strategies strike a balance between the optimum utilization of limited vaccine resources (which become gradually available and are subject to logistical and distribution constraints), on the one hand, and the deployment of NPIs. The objective is to minimize mortality rates together with the negative social and economic effects of prolonged NPIs. Several mathematical epidemiological models have been proposed in the literature to capture COVID-19 disease dynamics and assess control policies. These fall in two broad classes: deterministic and stochastic. Typical deterministic compartmental models are based on a set of differential equations describing transition dynamics between different compartments, and assume uniform (homogeneous) mixing of the population. A deterministic model will always yield the same output from a given initial condition. Alternatively, stochastic models incorporate the inherent stochasticity of infectious disease outbreaks and also account for heterogeneous mixing patterns of the affected population. Ιt is noted that all containment strategies work through networks, either perturbing (e.g., social distancing) or exploiting them (e.g., contact tracing), the need to model stochasticity, heterogeneity, and the structure of the population contact networks when studying COVID-19 transmission acquire critical importance. Stochastic models should thus be a natural choice to study the utility of specific NPIs or the accrued benefits of combining NPIs and pharmaceutical interventions (such as usage of vaccines).

In this paper we develop, implement and test a two-phase stochastic dynamic network compartmental model: First a pre-vaccination Susceptible-Exposed-Infected-Recovered (SEIR) until 15 February 2021, followed by a post-vaccination Susceptible-Vaccinated-Exposed-Infected-Recovered (SVEIR) which started on February 15th, 2021 and ended June 30th, 2021. Vaccination is combined with mid-term Non-Pharmaceutical Interventions (NPIs) to minimize human losses and to limit social and economic costs. Three scenarios are assessed for the first phase: (a) A baseline scenario lifting the national lockdown and all NPIs on 8 January 2021; (b) a “semi-lockdown” scenario with school opening on 8 January 2021, partial retail sector operating, universal mask wearing and social distancing/teleworking; and (c) a “rolling lockdown” scenario combining a partial lifting of measures on 8 January with a third imposed nationwide lockdown in February 2021. For the second phase three scenarios with different vaccination rates are assessed. Publicly available data along with some preliminary results of the SHARE COVID-19 survey for Greece are used as input (http://www.share-project.org/special-data-sets/share-corona-survey.html; accessed on 17 December 2020). The results are showcasing mid-term policy recommendations regarding the pandemic containment in Greece and could support decision makers in suggesting a policy pathway to be followed until the majority of the population is vaccinated.

The remainder of this paper is organized as follows. Section 2 provides a comprehensive review of available stochastic COVID-19 epidemiological models with a specific focus on studies incorporating vaccination-related strategies. In Section 3, the proposed two-phase stochastic model is described, whereas in Section 4, the experimental results of the simulation runs are presented. Finally, Section 5 discusses the benefits of the proposed model and its policy implications.

## 2. Background Literature

Beginning in the first days of 2020, COVID19-related epidemiological literature has thrived. Many models have been proposed to capture its transmission mechanism and characteristics [2,3]. Given that an effective vaccine was unavailable, most have focused on NPIs to control the COVID19 outbreak. Such interventions include quarantine over certain regions, social distancing, use of face masks, hand hygiene, etc. Of particular interest are stochastic epidemic models that, in contrast to deterministic models, capture disease transmission characteristics as well as the heterogeneity of population dynamics. Stochasticity in disease dynamics can be captured either by agent-based models [4,5] or by stochastic compartmental models [6,7].

The advent of COVID19-related vaccines has a significant impact on how control policies should be modelled and assessed (https://www.ecdc.europa.eu/en/publications-data/covid-19-vaccination-and-prioritisation-strategies-eueea; accessed on 23 December 2020). Two principal streams of vaccine-related papers within the COVID19 literature can be distinguished: The first addresses vaccine prioritization (across different age groups, regions, timeframes, etc.), minimum immunization thresholds for achieving herd immunity and types of immunization campaign undertaken (impulsive/voluntary vaccination, etc.). For example, in [8] the authors assess the optimal allocation of a limited vaccine supply across different age groups. They investigate three alternative policy objectives (minimizing infections, years of life lost, or deaths); they show how optimal prioritization is responsive to many parameters, most notably the efficacy and availability of vaccines, the rate of transmission and the severity of initial infections. A similar approach is followed in [9], where the authors use a mathematical model to compare five age-stratified prioritization strategies. An information-dependent model for assessing behavioral aspects of an immunization campaign is presented in [10]. The overall vaccination process is presumed to be completely voluntary, while it is assumed that the decision to get vaccinated or not depends in part on the information and rumors available about the spread of the disease in the population. In [11] the authors apply an age-stratified mathematical model to determine optimal vaccine allocation strategies depending on four different metrics (deaths, symptomatic infections and maximum non-ICU and ICU hospitalizations). An important aspect in designing immunization campaigns is the speed-versus-efficacy tradeoff. In [12] the authors compare the performance characteristics of one- and two-dose vaccine candidates over a six-month horizon on outcomes of cumulative infections, deaths, and peak hospitalizations. Other studies identify the necessary vaccine stockpile level to achieve herd immunity within a given population for different levels of a vaccine’s efficiency [13,14]. In [15] the authors suggest a time- and space-based vaccine distribution strategy that sequentially prioritizes regions with the most recent cases of infection within a certain time period; it contrasts it with the current practice of demographically distributing vaccines. The suggested vaccine distribution plan sets out the idea that individuals should be prioritized not only by individual characteristics, such as their risk of spreading the disease, but also by the area in which they reside. Two optimal control problems (single- and multi-objective) are suggested to assess a vaccine administration strategy for COVID-19 in [16]. The first strategy is to minimize the number of people infected during treatment whereas the second considers that the sum of affected individuals and the prescribed vaccine concentration should be reduced together during the control effort. Finally, in [17] the authors assess two vaccination strategies, newborns vaccination and voluntary vaccination.

The second literature stream focuses on the possible benefits of combining vaccination with NPIs such as surveillance, social distancing, social relaxation, quarantining, patient treatment/isolation, etc. with various levels of vaccine efficiency [18]. In [19] the authors construct a game-theoretic model for disease transmission and test two control measures, vaccination and social distancing. In [20] the authors examine the combination of immunization campaigns with other control measures such as the use of face masks and social distancing. Vaccination may prove critical for disease dynamics in the case of subpopulations extremely vulnerable to COVID19 (such as the elderly or institutionalized individuals). Such groups may give rise to hotspots driving infection within the general population [21]. Targeted vaccination of these groups may be sufficient to interrupt regional spread and protect a much wider fraction of the public. In [22] the authors identify the necessary number of vaccines and vaccine efficacy thresholds capable of preventing an epidemic whilst adhering to lockdown guidelines. Assuming a vaccine efficacy of 100% in a mass vaccination program, at least 60% of a given population should be vaccinated to obtain herd immunity. It should be noted that to eliminate disease transmission, a highly effective vaccine is required and, therefore, complementing vaccination with other interventions, such as face mask usage and/or social distancing will always yield optimal containment results [23].

The proposed two-phase (a pre-vaccination SEIR and a post-vaccination SVEIR) stochastic dynamic network based compartmental model is used to forecast the relative impact on COVID-19 pandemic of a set of major NPIs, which can be specified on a targeted individual basis, along with voluntary mass vaccination. It enables rigorous analysis of transmission patterns and realistic interventions based on the properties of networks. It is the first time since the pandemic’s outbreak that such a type of model is applied to yield a mid-term forecast in Greece.

## 3. The Model

### 3.1. Phase-1 Model

**Figure 1:**
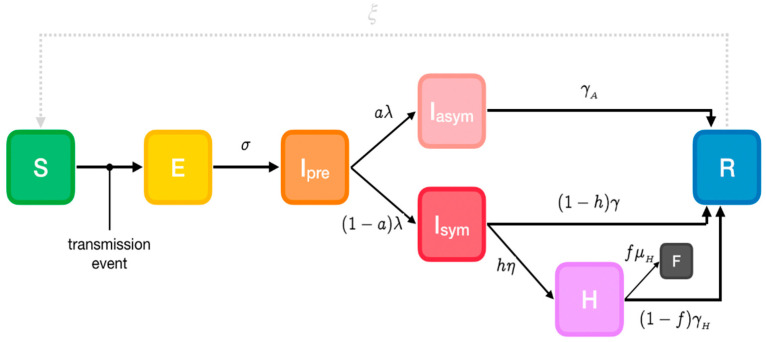
Phase-1 Susceptible-Exposed-Infectious-Recovered (SEIR) model. Cases are classified into susceptible (S), exposed (E), infectious (I, which is divided into 3 compartments: I_pre_, before developing symptoms and I_symp_ for clinically ill or I_asymp_ for true asymptomatic), Hospitalized (H), fatalities (F) and recovered (R) (https://github.com/ryansmcgee/seirsplus; accessed on 1 November 2020).

A modified version of the extended Susceptible-Exposed-Infectious-Recovered (SEIR) model (https://github.com/ryansmcgee/seirsplus; accessed on 1 November 2020) is used to represent SARS-CoV-2 disease states (Figure 1) in the first phase when pharmaceutical interventions (vaccines) are not available. Susceptible individuals (S) from a population of size N become exposed (E) if they make a transmissive contact with already infectious individuals. These newly exposed individuals first experience a latent period during which they are not infectious. Then they progress to a pre-symptomatic infectious state (I_pre_), when they are infectious but asymptomatic. A proportion develops symptoms (I_sym_), while the remainder never develop symptoms despite being infectious (I_asym_). A subset of symptomatic individuals progress to a more severe clinical state and must be hospitalized (IH), while a fraction of these severe cases are fatal (F). At the end of the infectious period, infected individuals enter the recovered state (R) and are no longer infectious or susceptible to infection. It must be noted that recovered individuals may become resusceptible sometime after recovering, though with a highly uncertain and in any case very low rate [24]. Transmissibility, rates of progression and other properties vary between the disease states.

#### Parameters

The model’s parameters are the following:

- *α*: Susceptibility
- *β*: Transmissibility of symptomatic individuals
- *β_A_*: Transmissibility of pre- and asymptomatic individuals
- *σ*: Rate of progression to infectiousness (1/latent period)
- *λ*: Rate of progression to (a)symptomatic state (1/pre-symptomatic period)
- *a*: Probability of an infected individual remaining asymptomatic
- *h*: Probability of a symptomatic individual being hospitalized
- *η*: Rate of progression to hospitalized state (1/onset-to-admission period)
- *γ*: Rate of recovery for non-hospitalized symptomatic individuals (1/symptomatic individuals infectious period)
- *γ_A_*: Rate of recovery for asymptomatic individuals (1/asymptomatic individuals infectious period)
- *γ_H_*: Rate of recovery for hospitalized symptomatic individuals (1/hospitalized individuals infectious period)
- *f*: Probability of death for hospitalized individuals (case fatality rate)
- *μ_H_*: Rate of death for hospitalized individuals (1/hospital admission to death period)
- *ξ*: Rate of re-susceptibility (1/temporary immunity period; equal to 0 if a permanent immunity period is assumed)

### 3.2. Phase-2 Model

**Figure 2:**
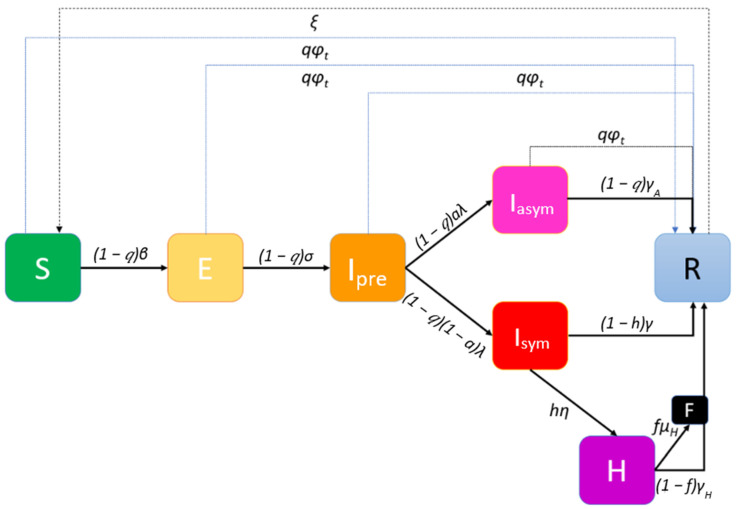
Phase-2 extended Susceptible-Vaccinated-Exposed-Infected-Recovered (SVEIR) model with a vaccination rate *φ_t_* at day *t*.

Worldwide vaccination, ongoing since end-December 2020, is to take place through hospitals, health centers and other primary health care infrastructures. Moreover, mobile medical teams will vaccinate people unable to proceed to vaccination centers, either for being house-bound (elderly, incapacitated etc.) or living in institutions. To illustrate this second phase of the pandemic, an extended Susceptible-Vaccinated-Exposed-Infected-Recovered (SVEIR) model is implemented (Figure 2).

The number of vaccines available at all vaccination centers at different discrete time periods (usually days) *t*, a parameter that will affect the course of the outbreak, is expressed via the vaccination rate *φ_t_*. Vaccination is voluntary and the vaccine will have an expected efficacy (if administered in a two-dose scheme, the efficacy starts with the reminder dose). This efficacy is assumed to be the same for preventing infection/transmission and for clinical disease; it is captured in the model by the proportion q of the population that will be willing to participate and at the same time the vaccine will have the desired effects. It is assumed that the vaccine is a “waning/all-or-nothing” one, meaning that some individuals will be granted perfect immunity for a period of time/permanently, but others will have zero protection. As all asymptomatic individuals may present themselves to the vaccination queue, the available vaccines might be allocated to those susceptible to the disease that are eligible for vaccination as well as to those that are in states *I_pre_* and *I_asym_*.

### 3.3. Stochastic Network Model Implementation

Let a graph G represent individuals (nodes) and their interactions (edges). Each individual (node) *i* is in a state *X_i_* ∈ {*S*, *E*, *I_pre_*, *I_sym_*, *I_asym_*, *H*, *R*, *F*}. The nodes adjacent (connected by a single edge) to an individual *i* define its set of “close contacts” *C_G_*_(*i*)_, representing individuals with whom there is a regular interaction, such as household members, co-workers, friends, etc. This set cardinality stands for the degree of the network. At any time, individuals make either random contacts in their set of close contacts with probability (1-*p*), or contact with individuals randomly sampled from the population at large, with probability *p*. These “global contacts” are one-off encounters with rare acquaintances or unknown individuals (for example at the supermarket, on public transportation means, at a social event, etc.). Probability p defines the “locality” of an individual’s contacts’ network: for *p* = 0 an individual only interacts with close contacts, while *p* = 1 represents a uniformly mixed population. Transmission between an individual and his/her global contacts is referred to as global transmission, and transmission between an individual and his/her close contacts is referred to as local transmission. Finally, it is of major interest to consider the effect of ongoing introductions of the disease from outside the population. For example, an individual may interact with an infectious tourist, therefore introducing an entirely new transmission chain.

### 3.4. Non-Pharmaceutical Interventions (NPIs)

Social distancing and related NPIs (lockdowns, stay-at-home orders, “cocooning” of certain demographics, etc.) can be implemented by specifying a contact network. Dynamic scenarios can be explored by changing the structure of the contact network during a simulation. All model parameters are assigned to each node on an individual basis (arbitrary parameter heterogeneity). Varying the parameters as well as setting network properties such as the mean number of adjacent nodes (“close contacts”), allows modelling the severity of NPIs. Social distancing interventions may increase the locality of the network (i.e., decrease p) and/or decrease local connectivity of the network (i.e., decrease its degree).

### 3.5. Model Equations

Let **1***_Xi_*_= *Z*_ be an indicator function, equal to 1 if the state of node *i* is *Z*∈ {*S*, *E*, *I_pre_*, *I_sym_*, *I_asym_*, *H*, *R*, *F*}; equal to 0 otherwise. Then the propensities of state transitions (i.e., expected time to transition) for node *i* in Phase-1 model are given by the following equations:

(1) $$\begin{array}{ll} P\left(X_{i}=E/\;X_{i}=S\right) & =\alpha_{i}(p_{i}\frac{\bar{\beta }{Ι}_{sym}+\bar{\beta_{A}}\left(I_{pre}+I_{asym}\right)}{N}+(1\\ & -p_{i})\frac{\sum_{j\in \left|C_{G}\left(i\right)\right|}\delta_{ji}\left(\beta_{ji}1_{X_{j}=I_{sym}}+\beta_{ji,A}1_{X_{j}\in \left\{I_{pre},\;I_{asym}\right\}}\right)}{\left|C_{G}\left(i\right)\right|})1_{X_{i}=S} \end{array}$$

(2) $$P\left(X_{i}=I_{pre}\;/\;X_{i}=E\right)=\sigma_{i}1_{X_{i}=E}$$

(3) $$P\left(X_{i}=I_{sym}\;/\;X_{i}=I_{pre}\right)=(1-a_{i})\lambda_{i}1_{X_{i}=I_{pre}}$$

(4) $$P\left(X_{i}=I_{asym}\;/\;X_{i}=I_{pre}\right)=\alpha_{i}\lambda_{i}1_{X_{i}=I_{pre}}$$

(5) $$P\left(X_{i}=R\;/\;X_{i}=I_{sym}\right)=(1-h_{i})\gamma_{i}1_{X_{i}=I_{sym}}$$

(6) $$P\left(X_{i}=H\;/\;X_{i}=I_{sym}\right)=h_{i}n_{i}1_{X_{i}=I_{sym}}$$

(7) $$P\left(X_{i}=R\;/\;X_{i}=I_{asym}\right)=\gamma_{i,A}1_{X_{i}={Ι}_{asym}}$$

(8) $$P\left(X_{i}=R\;/\;X_{i}=H\right)=\left(1-f_{i}\right)\gamma_{i,H}1_{X_{i}=H}$$

(9) $$P\left(X_{i}=F\;/\;X_{i}=H\right)=f_{i}\mu_{i,H}1_{X_{i}=H}$$

The propensities of state transitions for node *i* in Phase-2 model are given by the following equations:

(10) $$\begin{array}{ll} P\left(X_{i}=E/\;X_{i}=S\right) & =\left(1-q_{i}\right)\alpha_{i}(p_{i}\frac{\bar{\beta }{Ι}_{sym}+\bar{\beta_{A}}\left(I_{pre}+I_{asym}\right)}{N}+(1\\ & -p_{i})\frac{\sum_{j\in \left|C_{G}\left(i\right)\right|}\delta_{ji}\left(\beta_{ji}1_{X_{j}=I_{sym}}+\beta_{ji,A}1_{X_{j}\in \left\{I_{pre},\;I_{asym}\right\}}\right)}{\left|C_{G}\left(i\right)\right|})1_{X_{i}=S} \end{array}$$

(11) $$P\left(X_{i}=I_{pre}\;/\;X_{i}=E\right)=\left(1-q_{i}\right)\sigma_{i}1_{X_{i}=E}$$

(12) $$P\left(X_{i}=I_{sym}\;/\;X_{i}=I_{pre}\right)=\left(1-q_{i}\right)(1-a_{i})\lambda_{i}1_{X_{i}=I_{pre}}$$

(13) $$P\left(X_{i}=I_{asym}\;/\;X_{i}=I_{pre}\right)=\left(1-q_{i}\right)\alpha_{i}\lambda_{i}1_{X_{i}=I_{pre}}$$

(14) $$P\left(X_{i}=R\;/\;X_{i}=I_{sym}\right)=(1-h_{i})\gamma_{i}1_{X_{i}=I_{sym}}$$

(15) $$P\left(X_{i}=H\;/\;X_{i}=I_{sym}\right)=h_{i}n_{i}1_{X_{i}=I_{sym}}$$

(16) $$P\left(X_{i}=R/X_{i}=I_{asym}\right)=\left(1-q_{i}\right)\gamma_{i,A}1_{X_{i}={Ι}_{asym}}+q_{i}\varphi_{t}1_{X_{i}={Ι}_{asym}}$$

(17) $$P\left(X_{i}=R\;/\;X_{i}=H\right)=\left(1-f_{i}\right)\gamma_{i,H}1_{X_{i}=H}$$

(18) $$P\left(X_{i}=F\;/\;X_{i}=H\right)=f_{i}\mu_{i,H}1_{X_{i}=H}$$

(19) $$P\left(X_{i}=R\;/\;X_{i}=S\right)=q_{i}\varphi_{t}1_{X_{i}=S}$$

(20) $$P\left(X_{i}=R\;/\;X_{i}=E\right)=q_{i}\varphi_{t}1_{X_{i}=E}$$

### 3.6. Assumptions

- Regarding global interactions, every node in the population is equally likely to come into contact with every other node, and the population can be considered well-mixed. Thus, the contribution of the symptomatic subpopulation to an individual’s propensity for exposure is expressed through the mean transmissibility of the symptomatic individuals $\bar{\beta }$ and the prevalence of symptomatic individuals in the overall population *I_sym_*/*N*. The contribution of pre- and asymptomatic infectious individuals involves the mean transmissibility of asymptomatic individuals $\bar{\beta_{A}}$ and their prevalence (*I_pre_* + *I_asym_*)/*N* (https://github.com/ryansmcgee/seirsplus; accessed on 1 November 2020).
- Regarding local transmission, transmissibility is considered on a pairwise basis, i.e., every directed edge of the contact network representing transmission from infected node j to susceptible node i is assigned a transmissibility *β_ji_*. The transmissibility of such an interaction is assumed in the proposed model to be equal to the transmissibility of the infected individual (i.e., *β_ji_* = *β_j_*). The transition rate for any susceptible individual to become exposed due to local transmission is the product of that individual’s susceptibility and the total transmissibility of his/her infectious close contacts, divided by the size of his/her local network. The factor *δ_ji_* appears in the calculation of the propensity for exposure due to local transmission and is used to weight the transmissibility of interactions according to the connectivity of the interacting individuals, for example yielding a higher importance to a transmission between highly connected nodes-“superspreaders” (individuals who have more contacts also have more intense interactions). In this paper *δ_ji_* is defined as:
  (21) $$\delta_{ji}=\frac{logD_{j}+logD_{i}}{2log\bar{D}}$$
  where *D_j_* and *D_i_* are the degrees of nodes j and i, respectively, and $\bar{D}$ is the mean degree of the network. Similar to the global transmission, the contributions of symptomatic and pre-symptomatic infectious contacts to the propensity for exposure are calculated separately (https://github.com/ryansmcgee/seirsplus; accessed on 1 November 2020).
- The rate of re-susceptibility is assumed to be negligible (ξ is assumed to be equal to zero).
- The percentage q of the individuals that will be willing to participate in the mass vaccination and at the same time the vaccine will be effective on them is considered constant for all states.
- The number of vaccine-related deaths is negligible. This assumption is justified if individuals contraindicated for vaccination are totally excluded from the vaccination queue by a medical pre-screening.

## 4. Simulations’ Results

### 4.1. Covid-19 Background in Greece

In Greece, the first COVID-19 case was reported on 26 February 2020 and, soon after, numerous NPIs were implemented. A first nationwide lockdown took place from 23 March to 4 May 2020 and a second, less stringent, was imposed on 7 November 2020 and as of 31 December 2020 is still ongoing. A timeline of the major NPIs imposed in Greece is illustrated in Table 1.

**Figure 3:**
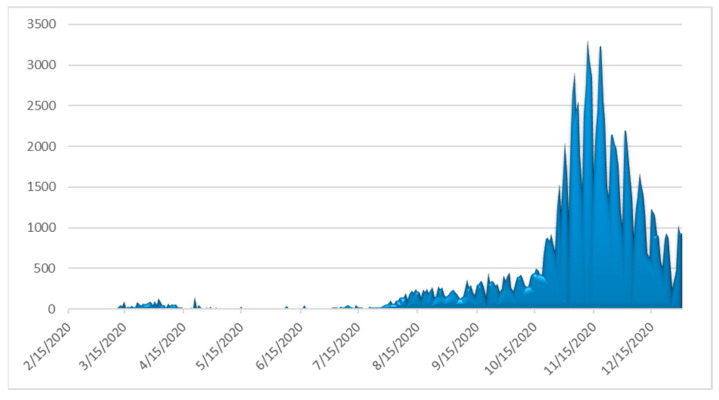
Daily number of laboratory-confirmed COVID-19 cases in Greece (data available from ECDC (https://www.ecdc.europa.eu/en/publications-data/covid-19-testing; accessed on 2 March 2021).

**Figure 4:**
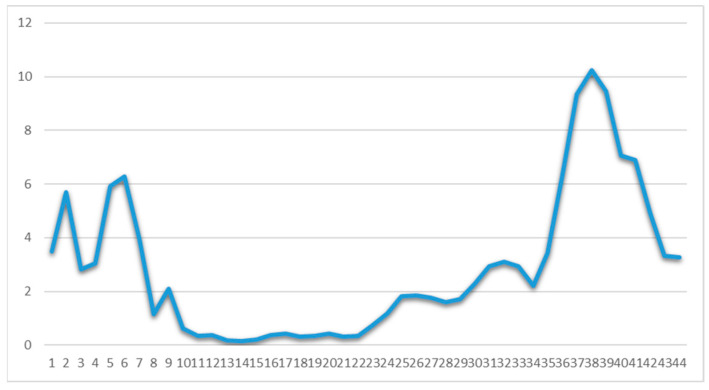
Weekly ratio (%) of laboratory-confirmed COVID-19 new cases to tests in Greece (Data available from ECDC (https://www.ecdc.europa.eu/en/publications-data/covid-19-testing; accessed on 2 March 2021). Week one corresponds to 24 February–1 March 2020.

**Figure 5:**
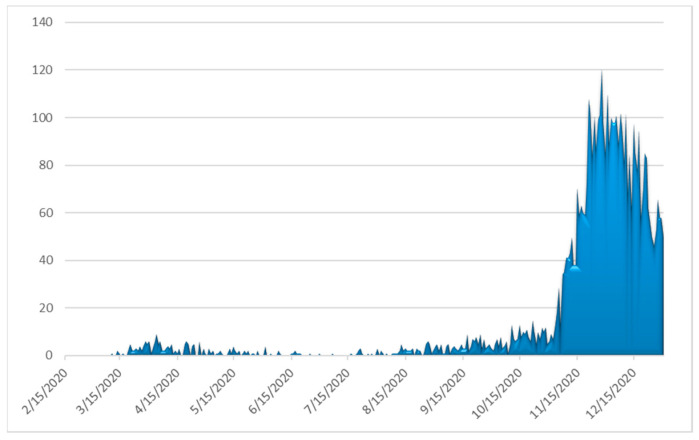
Observed daily confirmed number of deaths from COVID-19 complications in Greece (Data available from ECDC (https://www.ecdc.europa.eu/en/publications-data/covid-19-testing; accessed on 2 March 2021).

The number of daily confirmed cases by date of sampling for laboratory testing, the more informative weekly new cases to tests percentage ratio (“positivity”) and the daily number of deaths as of 31 December 2020 are depicted in Figure 3, Figure 4 and Figure 5, respectively. By 31 December 2020, there were 138,850 diagnosed cases and 4838 deaths. As of 31 December 2020, the corresponding naive case fatality rate in Greece was 3.48% and the mortality rate approximately 45 deaths per 100,000 population, ranking the country 126th out of 171 worldwide and 17th out of 50 Europe-wide (Mortality Analyses—Johns Hopkins Coronavirus Resource Center (jhu.edu); accessed on 31 December 2020).

### 4.2. Scenarios

First, the approximately 10,800,000 individuals in Greece were decade-wise categorized into nine age groups, from “0–9 year” to “80+ years”, owing to the strong age-dependence of COVID-19 progression and symptoms (lower attack rates and susceptibility among younger individuals and especially children) [25] and on the age-dependence of the contacts’ network structure (younger individuals may have 3–4 times more “global contacts” than older individuals). Given that the COVID-19 pandemic started in Greece less than a year ago, it is assumed that no changes in the population have occurred due to births, deaths, migration, etc.

The outbreak of SARS-CoV-2 in Greece was simulated using Phase-1 SEIR model from the initiation of transmission until 15 February 2021 and Phase-2 SVEIR model from 15 February 2021 to 30 June 2021. Transmission is deemed to have started on 15 February 2020, as the earliest reported date of symptom onset among infected cases was 20 February 2020, assuming that infection occurs approximately five days before (mean incubation period [25]). 15 February 2021 was selected for being exactly one year from the first recorded transmission; it also corresponds to the date by which the Greek government is planning to have achieved immunization through vaccination of approximately 1% of the total population (emvolio.gov.gr; accessed on 2 March 2021). It should be noted that the government plans to complete the mass vaccination of the population by 30 June 2021.

The Greek government allowed partial retail opening (“click away” mode) on 14 December 2020. Under this framework, three scenarios are assessed using the Phase-1 model until 15 February 2021:

- The *baseline scenario*, where the national lockdown and all NPIs are lifted on 8 January 2021;
- A “*semi-lockdown*” scenario with school opening on 8 January 2021, partial retail sector operating, universal mask wearing and at least 50% teleworking; and
- A “*rolling lockdown*” scenario with a partial measures’ lift on 8 January 2021 and a third imposed nationwide lockdown in February 2021 (“rolling lockdowns” policy).

The models’ parameter values estimates (either considered non age-dependent or adjusted to account for the non-homogeneous stratified age-groups distribution of the Greek population) are listed in Table 2 and Table 3, respectively. The size and relative frequency of the age groups are based upon the most recent official national census data from the Hellenic Statistical Authority and are illustrated in Table 3. Publicly available data along with some first results of the SHARE COVID-19 survey conducted in Greece (for more information see the Appendix A) are used as input. Specifically for the calculation of β, the estimation of COVID-19 basic reproduction number R_0_ in Greece ([25]), which is 2.38 (95% CI = [2.01, 2.8]), is used.

The major effects of NPIs over time on the rates of infection in the population in the context of the three assessed scenarios are captured as follows:

- *Social distancing/teleworking/school closure*. Social distancing/teleworking/school closure effect is captured on the one hand by the reduction of the individuals’ daily contacts (degree of the individual’s contact network), and on the other hand by the reduction of the locality parameter p. The graphs’ mean degrees and p by age group without any NPIs imposed are shown in Table 3 and the decrease in their values by age group when social distancing/teleworking/school closure measures are imposed is illustrated in Table 4.
- *Lockdown*. Lockdown stringency is captured with a further decrease in the individuals’ contacts network degree and in the probability p of individuals coming into contact with those outside their immediate network. This decrease differs by age group and the stringency of lockdown and is illustrated in Table 4.
- *Mask wearing*. The factor by which β is reduced as a result of mask wearing depends on the utilization level (simple recommendation/strong recommendation/mandatory) as well as individuals’ adherence, which is age-dependent. This decrease is illustrated in Table 4.

The Phase-2 model’s sizes of initial states are derived from the optimal scenario in terms of fatalities yielded from Phase-1 model.

Regarding the mass vaccination strategy, as of 31 December 2020 Greece had received two shipments of Pfizer/BioNTech Comirnaty of 93,600 vaccines in total. The plan is to receive from the Pfizer/BioNTech, Moderna and AstraZeneca companies 919,250 vaccines until 31 January 2021, 1,133,450 vaccines until 28 February 2021, 2,995,800 vaccines until 31 March 2021 and 2,200,000 vaccines until 30 April 2021. Additional quantities, either from these three companies or from others that are developing their own vaccines, are scheduled to become available around the end of Spring 2021 (emvolio.gov.gr; accessed on 31 December 2020).

The vaccination is to take place in 1018 centers (hospitals, health centers, and other primary health care infrastructure) distributed all over Greece, while 100 mobile units will vaccinate individuals who cannot access vaccination centers (house-bound or institutionalized population). The vaccination design capacity is 5 individuals/hour/center, with centers operating 16h/day, 6 days/week. This is translated to 80 individuals vaccinated/center/day or 2,117,440 individuals/month nationwide. The vaccination prioritization the Greek government has announced places health workers and institutionalized elderly individuals first, followed by individuals aged over 65 and individuals with health problems, after whom comes the remainder of the population.

The available vaccine from Pfizer/BioNTech requires a reminder dose 21 days after the first dose, and its efficacy (after the reminder dose has been administered) is estimated to be around 90% for the individuals that are eligible for vaccination [33]. By 15 February 2021, approximately 100,000 individuals are scheduled to be vaccinated, thus approximately 90,000 individuals will be immunized (moved to the recovered state). A 70% voluntary participation of the adult population is assumed based on statistical surveys conducted in Greece around mid-December 2020; with a 90% efficacy it is estimated that q = 63%.

Then three scenarios with different expected vaccination rates are assessed, depending on the value of φ_t_:

- An optimistic scenario with φ_t_ = 81,440 vaccinated persons per day (this is the design vaccination capacity).
- A most likely scenario with φ_t_ = 60,000 vaccinated persons per day
- A pessimistic scenario with φ_t_ = 40,000 vaccinated persons per day

The most likely and pessimistic vaccination rates estimations are based on the vaccines’ planned available quantities and some preliminary data regarding the mass vaccination campaign in Greece till mid-January 2021.”

### 4.3. Implementation

Phase-1 and Phase-2 models were built using the SEIRS+ modelling tool (https://github.com/ryansmcgee/seirsplus; accessed on 1 November 2020), version 1.0.9. The code in Python is available on GitHub (https://github.com/filipposfwt/UNIPI_COVID-19; accessed on 5 January 2021). The size of the total population was set to 10,800 (a representative typical case, that is a factor of 1000 from the population of approximately 10,800,000 [26]). One symptomatic node (1000 cases) was seeded to the population at day 0 (15 February 2020). The two-phase model yields results for 500 days (365 days for phase-1 and 135 days for the phase-2 model) and the epidemic was further calibrated every 1000 new infected cases over the first 9.5 months. One-hundred runs were performed for each scenario for both phases.

Default interaction networks were used, constructed as FARZ graphs and processes. Stochastic network dynamics are simulated using the Gillespie algorithm, which is a common and rigorous method for simulating stochastic interaction dynamics [34]. Finally, ongoing exogenous introductions of the disease (imported new cases, e.g., tourists and land workers) were inserted to the population by manually interfacing with the model code during a run.

### 4.4. Results of the Scenarios

**Figure 6:**
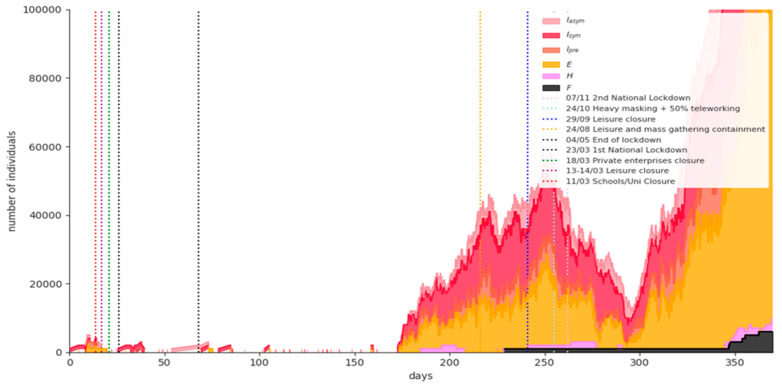
Simulation results for a representative phase-1 baseline scenario run.

Figure 6 shows the simulation results for a representative phase-1 baseline scenario run. Replacing the nationwide lockdown with only recommendations for social distancing measures on January 8th, 2021 results in uncontrolled disease spread. The expected fatalities on 15 February 2021 are 9920 (95% CI = [8372,11369]). It is obvious that this scenario is used only to prove that NPIs combined with vaccination are mandatory in order not to devastate the national health system.

**Figure 7:**
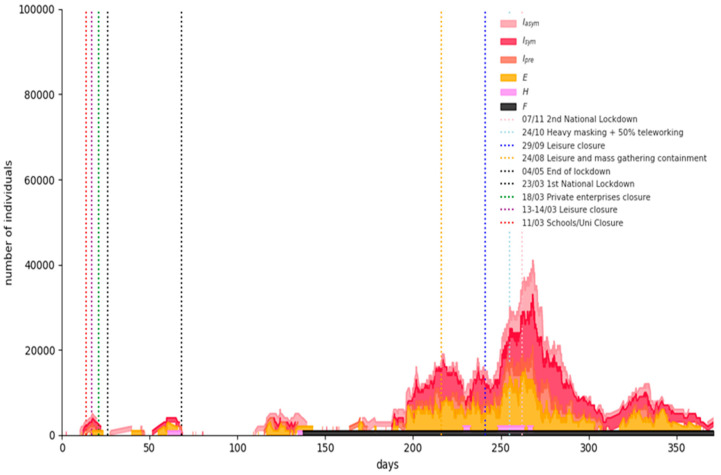
Simulation results for a representative Phase-1 “semi-lockdown” scenario run.

Figure 7 shows the simulation results for a representative Phase-1 “semi-lockdown” scenario run: universal masking and large-scale teleworking (at least 50%) flattens the curve significantly, although an increase in the numbers of infected and hospitalized individuals is evident due to the population mobility caused by schools’ opening and retailing partial operation. The expected fatalities on 15 February 2021 are 7530 (95% CI = [5948,9113]).

**Figure 8:**
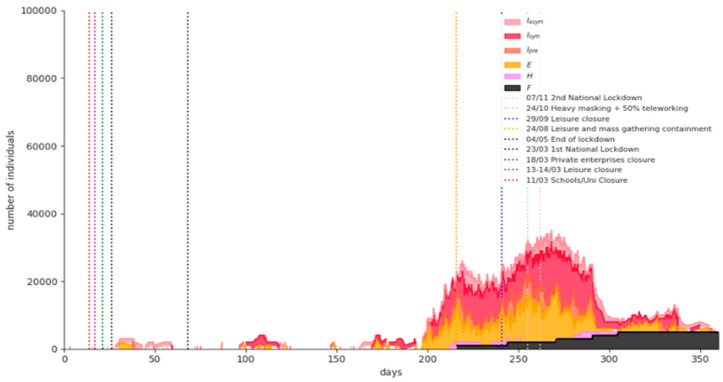
Simulation results for a representative Phase-1 “rolling lockdown” scenario run.

Figure 8 shows the simulation results for a representative phase-1 “rolling lockdown” scenario run: A partial measures’ lift scenario at 8 January 2021 (day 327) with a third imposed nationwide lockdown in February 2021. The expected fatalities on 15 February 2021 are 7960 (95% CI = [6350,9571]).

The results from the first phase indicate that the “semi-lockdown” scenario outperforms the “rolling” lockdown scenario (5.7% less expected fatalities), therefore, the phase-2 model’s initial states’ sizes are obtained from it. More particularly, the worst “semi-lockdown” scenario run ending states’ sizes were selected as initial states’ sizes for the SVEIR model. Conditional on this selection, a gradual lift of NPIs is placed on 1 April 2021.

**Figure 9:**
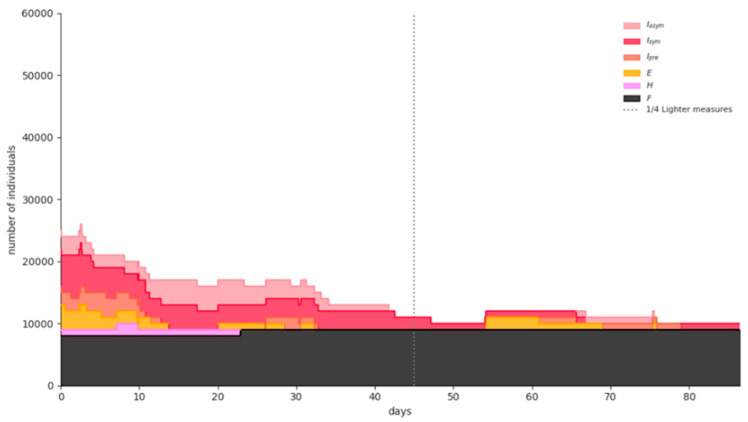
Simulation results for a representative phase-2 scenario run.

The simulation results for a representative run for any of the three different vaccination rates are similar to the ones illustrated in Figure 9. The expected number of fatalities from the beginning of the pandemic until 30 June 2021 for the optimistic, most likely and pessimistic scenarios are 8190 (95% CI = [8107,8274]), 8250 (95% CI = [8150,8350]), and 8290 (95% CI = [8200,8380]), respectively, indicating that the vaccines’ supply and vaccination rates are significant in minimizing fatalities from COVID-19, as is the case in all epidemics [35].

## 5. Discussion

The two-phase SEIR/SVEIR model proposed in this paper offers two key advantages in simulating COVID-19: First, given it is implemented on a stochastic dynamical network, it mimics interactions between individuals in society more closely; it avoids assuming uniform mixing as deterministic compartmental models do. Such an approach allows setting different model parameters for each node. Second, the NPIs and their introduction at different time periods are controlled in the simulation dynamically through the Python interface. This allows for policy interventions to be applied in response to possible changes in the spread of the epidemic (e.g., more stringent policies, such as a local or national lockdown, can be imposed when the number of new infections/prevalence rises above a threshold).

The phase-1 model suggests a substantial impact of universal masking and large-scale teleworking (at least 50%), i.e., an adequate containment of the number of new infections, with better results in terms of fatalities than a “rolling” lockdown policy. A hypothetical third stringent lockdown in February 2021 in Greece will eventually succeed to bring the disease under control. However, its economic and social impacts will be enormous, not to mention restrictions to individual freedoms. The two latter facts strongly argue for the adoption of an alternative solution, even retaining some restrictions such as leisure opening not before April 2021 and partial retail operating.

Regarding the model’s goodness of fit, at the end-date of the phase-1 model (15/2/21), the reported number of fatalities in Greece was 6152. This yields a percentage error of −18.3% from the expected number of fatalities in the “semi-lockdown” scenario, practically in the limits of the yielded 95% fatalities CI. The reason for this discrepancy is the 9-days nationwide lockdown imposed at the beginning of January 2021 and the local lockdown imposed in Athens since 11 February 2021, which were not included in the model during the simulations testing.

Regarding the phase-2 model, it is worth noting that fatalities are slightly different across the three vaccination scenarios in absolute figures. In fact, the people who will be vaccinated first until 31 March 2021, are the most vulnerable ones (individuals over 65 and/or individuals with health problems). Therefore, the number of hospitalized individuals and eventually, the fatality rate will be spectacularly reduced after April. Nevertheless, the difference of 100 fatalities between the optimistic and the pessimistic scenarios is considered as very significant in terms of human lives losses.

The search of an optimum strategy balancing efficacy with social and economic costs is not easy: a large number of minor NPIs applied in Greece from 4 May 2020 to the beginning of September 2020 did not have the results decision makers expected, and could not prevent the onset of the pandemic’s second and more destructive wave. On the other hand, fatigue of the population during the second national lockdown from November 2020 was partly responsible for its limited effectiveness compared to the first, which had been imposed in March 2020. This experience illustrates the salience of a mid-term decision support system for navigating a pathway to mass vaccination. The latter will eventually contain COVID-19, but the intervening period remains of great importance.

The analysis performed has several limitations. The lack of baseline pre-epidemic contact networks’ data implies that certain parameters’ values can only be guessed at. Even the use of SHARE COVID-19 survey collected in July 2020 for the over 50 population, could involve a recall bias, albeit unclear as to direction.

Additionally, the mass vaccination period raises many issues that are still not known:

- The vaccination prioritization is a very complex problem to solve. The vaccines that are gradually becoming available have different attributes, different administration schemes and different efficacies, varying from 62% to 94%.
- For the time being, the available data relate to the vaccines’ efficacy in preventing clinical disease only and not in halting infection/transmission. Moreover, in the proposed model efficacy is assumed to start as soon as the person is vaccinated in a single dose administration scheme (or after the reminder dose in a two-dose administration scheme), while a vaccinated individual might be still infectious for several days. The assumption seems reasonable under the light of the most recent research, where it seems that even after the first dose of vaccines administered in a two-dose scheme in a three-week time interval, efficacy is very high, reaching and exceeding 90% during days 15–28 after the first dose [36,37].
- The vaccines are considered as “waning/all-or-nothing” (providing perfect protection to a fraction of individuals who receive it). However, they could be proved to be “leaky”, i.e., only reducing the probability of infection. The proposed model can address this by calibrating accordingly parameter q.
- Finally, though a form must be filled by the individual before vaccination, this is unlikely to provide all the necessary information; vaccinators will be, for instance, to a large extent unaware of the susceptible/infectious status of all the asymptomatic individuals arriving at the vaccination system; it is, thus, certain that there will be a quantity of vaccines wasted per period. Therefore, an overestimation of vaccination results is possible in the proposed model.

## 6. Conclusions

Concluding, the close monitoring of COVID-19 is mandatory in order to adopt NPIs along with the mass vaccination campaign. Capturing social networks attributes and the population adherence to NPIs through repeated large-scale surveys is key to feed decision support tools with the proper data—if the pandemic is to be contained in 2021.

## Figures and Tables

**Table 1:** Major NPIs implemented in Greece during the COVID-19 pandemic.

Major NPIs	Starting Dates
School and University closures	11 March 2020
Leisure closure	13–14 March 2020
Private enterprises closure	18 March 2020
1st national lockdown	23 March 2020
Masking recommendations (“Light masking”)	4 May 2020
Leisure—mass gathering containment	24 August 2020
Leisure closure	29 September 2020
Mandatory masking (“Heavy masking”)	24 October 2020
2nd national lockdown	7 November 2020
Nursery schools—kindergarten—primary schools’ closure	14 November 2020

**Table 2:** Non age-dependent parameters’ values of the Phase 1 SEIR and Phase-2 SVEIR models used to assess COVID-19 transmission dynamics in Greece.

Parameter	Description	Value	Source
***β***	transmissibility of symptomatic individuals	[0.155, 0.4]	[25,26] and authors’ estimations
***β_A_***	transmissibility of pre- and asymptomatic individuals	*β/2*	
***σ***	rate of progression to infectiousness	(3.5 days)^−1^	
***λ***	rate of progression to (a)symptomatic state	(1.5 days)^−1^	
***n***	rate of progression to hospitalized state	(5.5 days)^−1^	
***γ***	rate of recovery for non-hospitalized symptomatic individuals	Gamma distributed with mean = (6 days)^−1^, s.d. = (2.4 days)^−1^	
***γ_A_***	rate of recovery for asymptomatic individuals	Gamma distributed with mean = (6 days)^−1^, s.d. = (2.4 days)^−1^	
***γ_H_***	rate of recovery for hospitalized symptomatic individuals	Gamma distributed with mean = (11.5 days)^−1^, s.d. = (2.3 days)^−1^	
***μ_H_***	rate of death for hospitalized individuals	Gamma distributed with mean = (11.774 days)^−1^, s.d. = (8.815 days)^−1^	

**Table 3:** Age-dependent parameters’ values used of the Phase 1 SEIR and Phase-2 SVEIR models used to assess COVID-19 transmission dynamics in Greece.

Age Group	Group Size (Hellenic Statistical Authority (statistics.gr; accessed on 10 November 2020))	Relative Frequencies (%)	Relative Susceptibility α [27]	% Infected Asymptomatic a [27,28]	% Hospitalization h [27,29] and Authors’ Estimations	%CFR for Hospitalized f [27,29] and Authors’ Estimations	Masking Adherence (%) [27] and Statistical Surveys	Social Distancing Adherence (%) [27] and Statistical Surveys	Mean Degree [22,26,30], Seirs+ Covid-19 Notebooks and Authors’ Estimations	p [31] and Authors’ Estimations
0 to 9	1,049,839	9.706	0.35	0.456	0.000011	0.0165	70–95	70–95	12.62	0.9
10 to 19	1,072,705	9.917	0.69	0.412	0.000114	0.0125			15.38	0.95
20–29	1,350,868	12.489	1.03	0.37	0.000495	0.025			12.89	0.7
30–39	1,635,304	15.119	1.03	0.332	0.003726	0.025			12.89	0.9
40–49	1,581,095	14.618	1.03	0.296	0.019272	0.0315			12.27	0.9
50–59	1,391,854	12.868	1.03	0.265	0.037107	0.08418	82.2	92.2	11.64	0.9
60–69	1,134,045	10.485	1.27	0.238	0.07067	0.1781	82.1	90.9	9.42	0.6
70–79	1,017,242	9.405	1.52	0.214	0.102833	0.38016	79.2	91.3	7.2	0.5
80+	583,334	5.393	1.52	0.192	0.131513	0.709	82.1	93.4	7.2	0.5

**Table 4:** Major NPIs effects on the model parameters’ values used of the Phase-1 SEIR and Phase-2 SVEIR models used to assess COVID-19 transmission dynamics in Greece.

Major NPIs	Starting Dates	Network Mean Degree (Age Distribution) [25,30] and Authors Estimations	*β* (Age Distribution) [26,32] and Authors Estimations	*p* (Age Distribution) [26,31] and Authors Estimations
School and University closures	11/3/2020	0–9: 6, 10–19: 12, 20–29: 11, 30–39:12, 40–49: 12, 50–59: 11, 60–69: 9, 70+: 7		0–19: 0.5, 20–39: 0.6, 40–69: 0.3, 70+: 0.2
Leisure closure	13–14/03/2020	0–9: 3, 10–19: 3, 20–29: 7, 30–39: 6 40–49: 6, 50–59: 6, 60–69: 8, 70+: 7		0–19: 0.3, 20–39: 0.4, 40–69: 0.3, 70+: 0.2
Private enterprises	18/03/2020	0–19:3, 20–29:6, 30–59: 5, 60–69: 6, 70+: 4		
1st national lockdown	23/03/2020	0–19: 3, 20–29: 5, 30–69: 3, 70+: 2		20–29: 0.05, Remainder: 0.02
“Light” masking	4/5/2020		0–49: 0.95β, 50+: 0.8β	
Leisure—mass gathering containment	24/08/2020	0–9: 12.62, 10–19: 15.38, 20–29: 12.89, 30–39:12.89, 40–49: 12.27, 50–59: 11.64, 60–69: 9.42, 70+: 7.2		
Leisure closure	29/09/2020	0–9: 12.62, 10–19: 15.38, 20–29: 12, 30–39:11, 40–49: 11, 50–59: 11, 60–69: 8, 70+: 7		0–19: 0.6, 20–39: 0.7, 40–69: 0.5, 70+: 0.3
«Heavy” masking +teleworking 50%	24/10/2020	0–9: 13, 10–19: 16, 20–29: 12, 30–39: 10, 40–49: 10, 50–59: 10, 60+: 7	0–19: 0.5β, 20–50: 0.65β, 50+: 0.2β	0–19: 0.5, 20–39: 0.6, 40–69: 0.4, 70+: 0.2
2nd national lockdown	7/11/2020	0–9: 12.62, 10–19: 7, 20–29: 8, 30–69: 7, 70+: 3		0–9: 0.3, 10–19: 0.3, 20–29: 0.1, Remainder: 0.03
Nursery schools—kindergarten—primary schools closure	14/11/2020	0–19: 4, 20–29: 7, 30–69: 5, 70+: 3		20–29: 0.1, Remainder: 0.03

**Figure A1:**
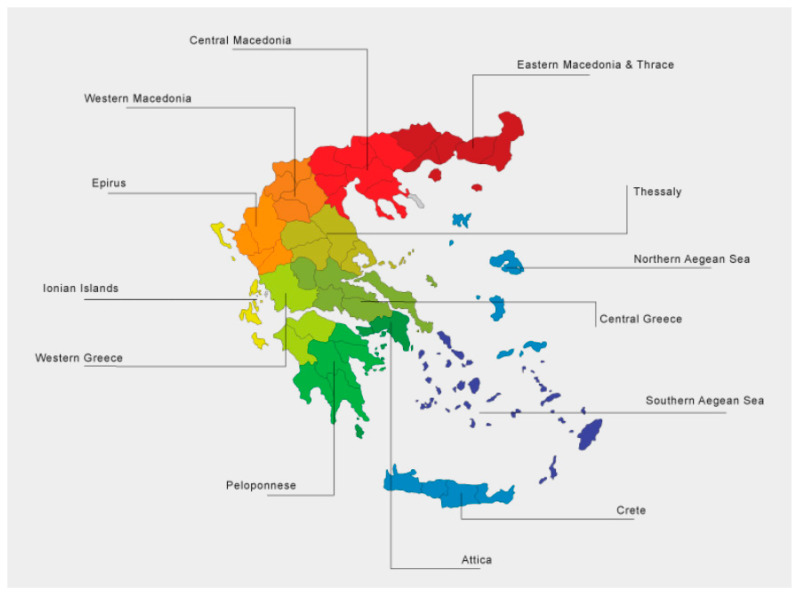
Map of Greece’s 13 administrative districts.

## Appendix A. The SHARE COVID-19 Survey

The Survey of Health, Ageing and Retirement in Europe (SHARE) (www.share-project.org; accessed on 17 December 2020) is a multidisciplinary and cross-national panel database of micro data on health, socio-economic status, and social and family networks of about 140,000 individuals aged 50 or older (around 380,000 interviews). SHARE covers 27 European countries and Israel. As a reaction to the seriousness of the COVID-19 outbreak and the prolonged worldwide lockdowns, a special SHARE COVID-19 questionnaire was developed. This new questionnaire covers the most important life domains for the target population and asks specific questions about:

- Health and health behavior (General health before and after the COVID-19 outbreak, practice of safety measures, e.g., social distancing, wearing a mask);
- Mental health (Anxiety, depression, sleeping problems, and loneliness before and after the COVID-19 outbreak);
- Infections and healthcare (COVID-19 related symptoms, SARS-CoV-2 testing and hospitalization, forgone medical treatment, satisfaction with treatments);
- Changes in work and economic situation (Unemployment, business closures, working from home, changes in working hours and income, financial support); and
- Social networks (Changes in personal contacts with family and friends, help given and received, personal care given and received).

In Greece, 3901 SHARE’s panel respondents (57% females) were interviewed via a Computer Assisted Telephone Interview (CATI) from June 12th to August 7th, 2020, answering the COVID-19 questionnaire. The sampling scheme was developed by the Hellenic Statistical Authority. The geographical distribution in Greece’s 13 administrative districts (Figure A1) and their ages’ relative frequencies’ distribution are illustrated in Table A1 and Table A2, respectively.

The COVID-19 questionnaire is available online (http://www.share-project.org/fileadmin/pdf_questionnaire_COVID-19/SHAREw8_COVID19_qnn_20200602_routing.pdf; accessed on 17 December 2020) [38]. Two questions were of particular interest for feeding the model, the one regarding wearing masks adherence and the one regarding social distancing adherence. Percentages were derived for 10-year age groups. The respondents’ answers are incorporated in Table 3.

**Table A1:** SHARE COVID-19 survey respondents’ geographical distribution.

District	Number of Participants in SHARE COVID-19 Survey
East Macedonia and Thrace	251
Central Macedonia	689
West Macedonia	132
Epirus	106
Thessaly	312
West Greece	200
Central Greece	228
Attica	1357
Peloponessos	192
Ionian islands	89
North Aegean	79
South Aegean	81
Crete	185

**Table A2:** Age distribution of SHARE COVID-19 survey in Greece.

Age	Relative Frequency
50–59 years old	13.6%
60–69 years old	35.8%
70–79 years old	30.1%
More than 80 years old	20.5%
